# Hospital-Based Contact Tracing of Patients With COVID-19 and Health Care Workers During the COVID-19 Pandemic in Eastern India: Cross-sectional Study

**DOI:** 10.2196/28519

**Published:** 2021-10-21

**Authors:** Durgesh Prasad Sahoo, Arvind Kumar Singh, Dinesh Prasad Sahu, Somen Kumar Pradhan, Binod Kumar Patro, Gitanjali Batmanabane, Baijayantimala Mishra, Bijayini Behera, Ambarish Das, G Susmita Dora, L Anand, S M Azhar, Jyolsna Nair, Sasmita Panigrahi, R Akshaya, Bimal Kumar Sahoo, Subhakanta Sahu, Suchismita Sahoo

**Affiliations:** 1 Department of Community Medicine and Family Medicine All India Institute of Medical Sciences, Bhubaneswar Odisha India; 2 All India Institute of Medical Sciences, Bhubaneswar Odisha India; 3 Department of Microbiology All India Institute of Medical Sciences, Bhubaneswar Odisha India; 4 College of Nursing All India Institute of Medical Sciences, Bhubaneswar Odisha India

**Keywords:** COVID-19, SARS-CoV-2, risk categorization, health care personnel, virus transmission, contact tracing, pandemic, risk stratification

## Abstract

**Background:**

The contact tracing and subsequent quarantining of health care workers (HCWs) are essential to minimizing the further transmission of SARS-CoV-2 infection and mitigating the shortage of HCWs during the COVID-19 pandemic situation.

**Objective:**

This study aimed to assess the yield of contact tracing for COVID-19 cases and the risk stratification of HCWs who are exposed to these cases.

**Methods:**

This was an analysis of routine data that were collected for the contact tracing of COVID-19 cases at the All India Institute of Medical Sciences, Bhubaneswar, in Odisha, India. Data from March 19 to August 31, 2020, were considered for this study. COVID-19 cases were admitted patients, outpatients, or HCWs in the hospital. HCWs who were exposed to COVID-19 cases were categorized, per the risk stratification guidelines, as high-risk contacts or low-risk contacts

**Results:**

During contact tracing, 3411 HCWs were identified as those who were exposed to 360 COVID-19 cases. Of these 360 cases, 269 (74.7%) were either admitted patients or outpatients, and 91 (25.3%) were HCWs. After the risk stratification of the 3411 HCWs, 890 (26.1%) were categorized as high-risk contacts, and 2521 (73.9%) were categorized as low-risk contacts. The COVID-19 test positivity rates of high-risk contacts and low-risk contacts were 3.8% (34/890) and 1.9% (48/2521), respectively. The average number of high-risk contacts was significantly higher when the COVID-19 case was an admitted patient (number of contacts: mean 6.6) rather than when the COVID-19 case was an HCW (number of contacts: mean 4.0) or outpatient (number of contacts: mean 0.2; *P*=.009). Similarly, the average number of high-risk contacts was higher when the COVID-19 case was admitted in a non–COVID-19 area (number of contacts: mean 15.8) rather than when such cases were admitted in a COVID-19 area (number of contacts: mean 0.27; *P*<.001). There was a significant decline in the mean number of high-risk contacts over the study period (*P*=.003).

**Conclusions:**

Contact tracing and risk stratification were effective and helped to reduce the number of HCWs requiring quarantine. There was also a decline in the number of high-risk contacts during the study period. This indicates the role of the implementation of hospital-based, COVID-19–related infection control strategies. The contact tracing and risk stratification approaches that were designed in this study can also be implemented in other health care settings.

## Introduction

With 44 million confirmed cases and over 1 million confirmed deaths affecting all countries across the world, the COVID-19 pandemic is currently the largest pandemic of the century [1]. As of August 31, 2020, 35 million COVID-19 cases and 0.06 million deaths have been reported from India [2].

By September 17, 2020, countries reported to the World Health Organization (WHO) that 14% of COVID-19 cases were health care workers (HCWs) [3]. SARS-CoV-2 infection among HCWs not only poses the risk of infection to their family members, thus contributing to community spread, but also poses this risk to other HCWs and patients. Thus, apart from stringent infection prevention and control practices for reducing the exposure to infection, the contact tracing and subsequent quarantining of HCWs are essential to minimizing further transmission. Consequently, isolation after SARS-CoV-2 infection and quarantine following exposure to a confirmed case of COVID-19 can adversely reduce the availability of human resources. To mitigate the shortage of staff in hospitals, the WHO and Centers for Disease Control and Prevention (CDC) have given recommendations for stratifying the risk following exposure into 2 categories—low-risk exposure and high-risk exposure [4,5]. The Ministry of Health and Family Welfare (MoHFW) of the Government of India has also adopted these guidelines [6].

Contact tracing is a time- and resource-intensive exercise for community settings as well as hospital settings. However, it is one of the most important methods for infectious disease prevention. In our hospital, we used different methods that were described in literature to identify people who were exposed to COVID-19 cases, like the use of closed-circuit television (CCTV) footage and duty rosters and the passive reporting of contacts by departments and via telephonic inquiry. Contact tracing by using data extracted from administrative and clinical databases, such as electronic medical records, or by using CCTV footage (a real-time locating system) has been reported previously [7,8]. Although conventional contact tracing via continuous direct observation has been considered to be the gold-standard method for accurately quantifying contact time, it requires intensive human resources and is not cost-effective [9]. Self-reporting methods can be used as alternatives to direct observation due to the lower intensity of their human resource demands; however, there is a chance of bias compromising the accuracy of the data [10].

Although the processes of contact tracing, risk stratification, and quarantine may help to reduce the transmission of infection, it is not clear whether these processes help with reducing staff shortages in an already overwhelmed health system of a resource-constrained setting. A systematic review of 22 studies concluded that an integrated strategy for contact tracing, screening, quarantine, and isolation has the potential to reduce the incidence of SARS-CoV-2 infection [11]. However, most of the studies included in this systematic review were community based. The few studies that were conducted in a health facility setting suggested using working shifts and integrating infection control practices to reduce the number of infections in health care settings [12,13]. Unlike those in community settings, very aggressive contact tracing and quarantine policies for HCWs in health care settings may be challenging to implement due to the need to balance infection control and staff shortages [14-16]. A study from India reported the beneficial effect that stratification has on minimizing staff shortages resulting from unnecessary quarantine [17]. As there is a limited amount of literature available from such settings, our study may provide information on making public health decisions in a health care setting.

In our hospital, which caters to both patients with COVID-19 and other patients, we adopted the contact tracing and risk stratification approaches described by the WHO, CDC, and MoHFW to categorize COVID-19–exposed HCWs as high-risk contacts or low-risk contacts. This study was conducted to assess the yield of hospital-based contact tracing for patients and HCWs who tested positive for COVID-19 and the risk stratification of COVID-19–exposed HCWs in the hospital—a statutory body under the aegis of the MoHFW of the Government of India. We also compared the risk categorizations of different areas (COVID-19 and non–COVID-19 areas) and different categories of index cases (outpatient department [OPD], inpatient department [IPD], and HCW cases) to assess the variations.

## Methods

### Study Design

This study was a process evaluation of our routine contact tracing and risk stratification mechanisms at the study site. Data from March 19 to August 31, 2020, were collected.

### Study Site

This study was conducted at the All India Institute of Medical Sciences (AIIMS), Bhubaneswar, which is a 960-bed tertiary care teaching hospital located in Bhubaneswar, the capital city of Odisha (an eastern state of India).

### COVID-19–Related Clinical Services at the Study Site

**Figure 1:**
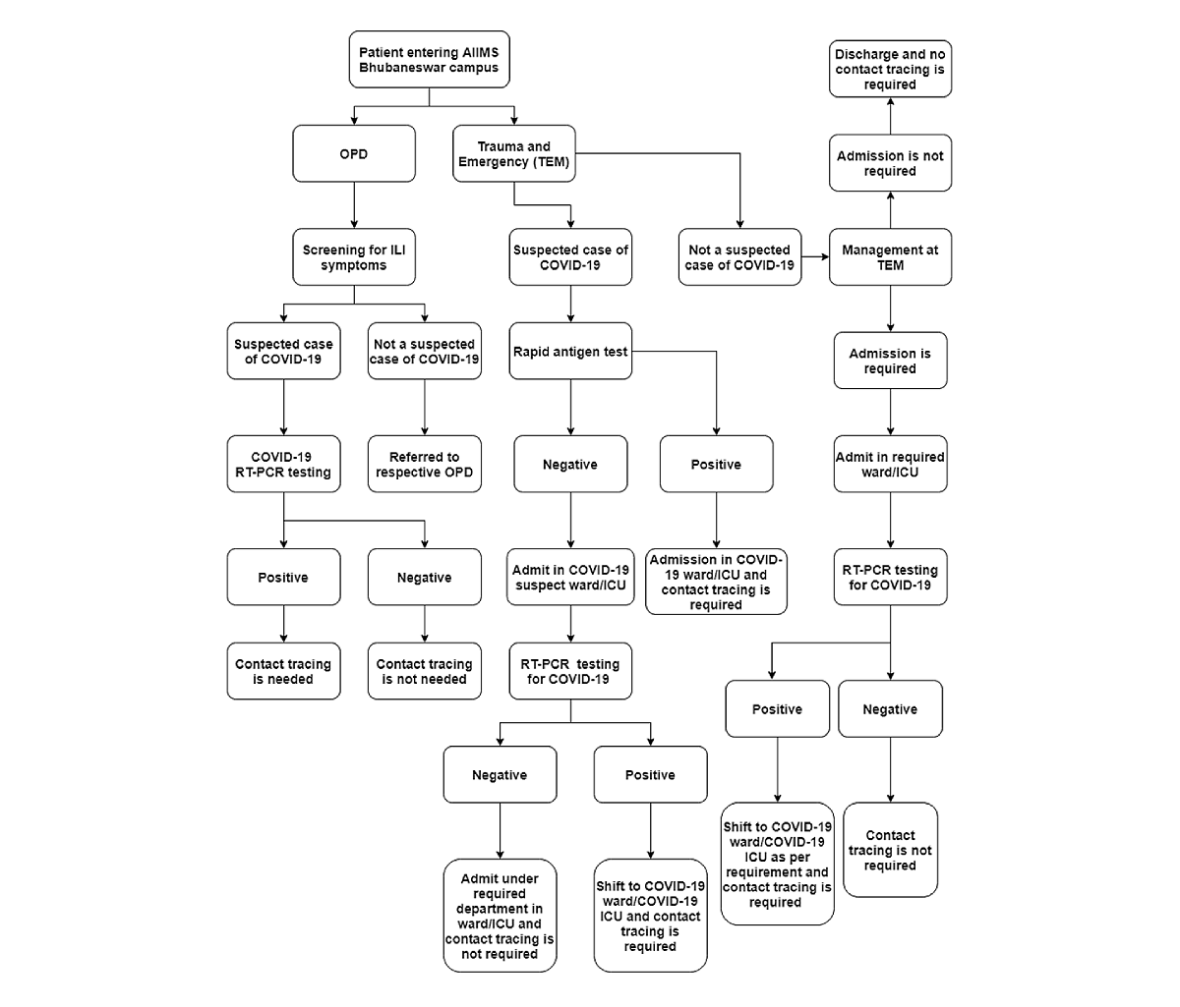
Algorithm of the COVID-19 testing strategy for patients admitted to the hospital. AIIMS: All India Institute of Medical Sciences; ICU: intensive care unit; ILI: influenza-like illness; OPD: outpatient department; RT-PCR: reverse transcription-polymerase chain reaction; TEM: trauma and emergency.

Patients who were admitted to the hospital were screened for COVID-19, as per the screening algorithm depicted in Figure 1. On March 19, 2020, the first patient (the second COVID-19 case of Odisha) with COVID-19 was admitted to our hospital. The first case of COVID-19 among HCWs was reported on June 2, 2020. COVID-19 screening via reverse transcription-polymerase chain reaction (RT-PCR) tests of all newly admitted patients, irrespective of the presence of symptoms, started on June 15, 2020. From July 10 onward, routine outpatient consultations were discontinued due to a sudden surge in the number of COVID-19 cases in the community and hospital. Hospital admission was restricted to only patients with COVID-19 and patients requiring emergency or essential intervention.

### COVID-19–Related Prevention Interventions at the Study Site

Various training programs were conducted to train all cadres of HCWs in the proper use of personal protective equipment (PPE), hand hygiene measures, and other infection control practices. The use of various types of PPE in different clinical areas and hospital premises was guided by MoHFW protocols and upgraded or modified based on feedback from the contact tracing and infection control teams. Advisories were issued to all HCWs at periodic intervals for PPE compliance and infection control measures. We also introduced various behavioral and regulatory interventions to promote COVID-19–appropriate behaviors, such as a monetary penalty for not using a mask in the hospital and residential campuses.

### Contact Tracing and Risk Stratification

**Figure 2:**
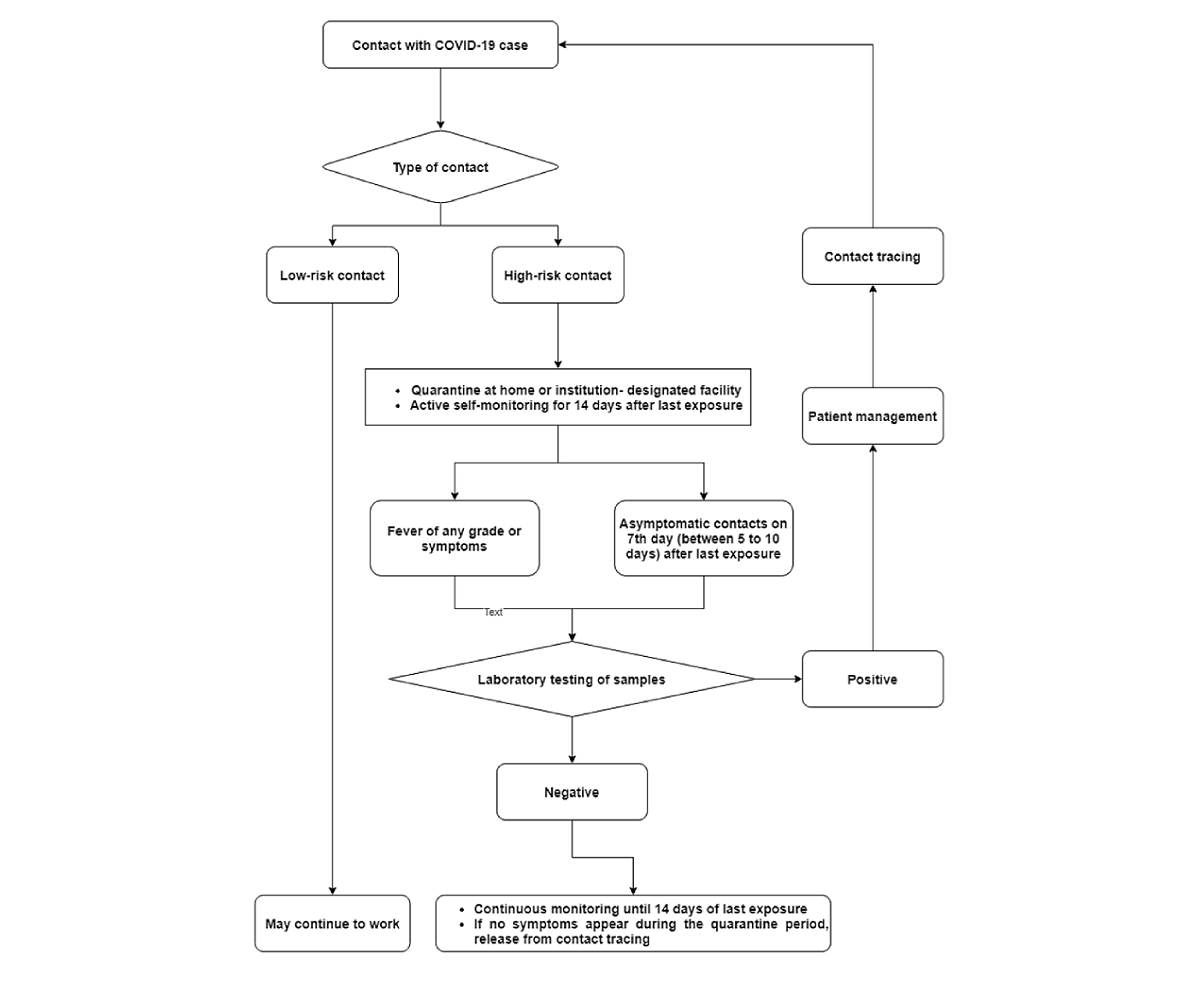
SARS-CoV-2 testing strategy for the health care workers after contact tracing.

As per the testing strategy outlined in Figure 2, testing for SARS-CoV-2 infection via the RT-PCR method was performed at the COVID-19 RT-PCR testing laboratory of the institute—an approved laboratory of the Indian Council of Medical Research of the Government of India. Patients presenting with symptoms that were consistent with COVID-19 and confirmed cases of COVID-19 were admitted in separate wards, which were referred to as *COVID-19 areas*. Patients with COVID-19 were categorized as inpatients, outpatients, or HCWs who tested positive for COVID-19. Inpatients were further categorized based on the area in which they were admitted (ie, COVID-19 areas or non–COVID-19 areas).

Contact tracing was initiated by a team that was dedicated to performing contact tracing immediately after intimation from the diagnostic laboratory. Initially, contact tracing was done by physically visiting the clinical areas, personally interviewing the HCWs involved in patient care, reviewing medical records of patients and duty rosters, and viewing CCTV footage. However, this strategy was modified, due to the increase in the number of COVID-19 cases, to include a passive mechanism for contact tracing. In the later phase (from July 15 onward), the contact tracing team (CTT) directed the concerned departments to provide a list of all HCWs who had possibly come in contact with confirmed COVID-19 cases in a prescribed format. Upon obtaining the list of COVID-19–exposed HCWs, the CTT contacted each HCW telephonically to elicit histories related to the durations and types of exposures, the procedures performed on the patient, and the use of PPE during exposures. Data were collected by using a semistructured interview schedule. For cases of contact tracing related to an HCW who tested positive for COVID-19, histories related to interactions that occurred during duty break hours, during meals, and in places where HCWs are likely to be less cautious in terms of mask usage were probed during contact tracing. Exposures the occurred during the last 14 days from the date of a positive report were considered for contact tracing. The numbers of contacts were separately calculated for each positive case.

Risk categorizations (low-risk exposure and high-risk exposure) based on the criteria adopted from the WHO, CDC, and MoHFW guidelines are given in Textbox 1. A 14-day home quarantine and COVID-19 testing, which was to be conducted on the seventh day after an HCW’s most recent exposure, were recommended for HCWs with high-risk exposures, whereas HCWs with low-risk exposures were recommended to continue their work. The quarantine period was considered to be a fully paid, on-duty period. Both risk categories were required to monitor symptoms and report on COVID-19 tests that were performed upon the appearance of symptoms consistent with COVID-19. In the absence of symptoms, routine testing was not recommended for low-risk contacts. However, a few HCWs with low-risk exposures were also tested upon their request. We collated data related to contact tracing and risk categorization in an Excel spreadsheet, and follow-ups of HCWs were done to inquire about symptoms and test results. The CTT regularly updated hospital authorities about their findings related to breaches in infection control practices and areas of high-risk contact and suggested specific recommendations.

**Risk categorization (low-risk exposure and high-risk exposure) of the health care workers who were exposed to patients who tested positive for COVID-19. The criteria were adopted from the World Health Organization, Centers for Disease Control and Prevention, and Ministry of Health and Family Welfare guidelines.**

**High-risk contact**

- Touched body fluids of a patient (eg, touching respiratory tract secretions, blood, vomit, saliva, urine, and feces; being coughed on; touching used paper tissues with a bare hand; etc)
- Had direct physical contact with the body of a patient, including during physical examinations without personal protective equipment
- Touched or cleaned the linens, clothes, or dishes of a patient
- Lives in the same household as a patient
- Anyone who was in close proximity (within 1 meter) to a confirmed COVID-19 case and did not take precautions
- Passengers (ie, those in a vehicle) who were in close proximity (for more than 6 hours) to a symptomatic person who later tested positive for COVID-19

**Low-risk contact**

- Shared the same space (worked in same room or a similar situation) but did not have a high-risk exposure to a confirmed case of COVID-19
- Traveled in the same environment (bus, train, flight, or any other mode of transit) but did not have a high-risk exposure

Ethical approval to conduct this study was obtained from the Institutional Ethics Committee of AIIMS, Bhubaneswar (reference number: T/IM-NF/CMFM/20/76). Individual participant consent was not obtained, as contact tracing was a regular process for risk stratification among the HCWs. All HCWs were instructed by the hospital authorities to cooperate with the CTT.

### Statistical Analysis

Statistical analyses were conducted by using Microsoft Excel 2013 and SPSS version 22.0 (IBM Corporation). Descriptive statistics were presented as means with SDs and percentages with 95% CIs. The mean number and SD of high-risk contacts and low-risk contacts among the types of patients (ie, admitted patients in a COVID-19 area, admitted patients in a non–COVID-19 area, outpatients, and HCWs) were compared. A *P* value of <.05 was considered statistically significant. We also compared the mean number of high-risk and low-risk contacts for a block of 15 days within the study period.

## Results

Our analysis included data related to 360 COVID-19 cases that were reported during the study period, which included 240 (66.7%) admitted patients and IPD patients, 29 (8.1%) OPD patients, and 91 (25.3%) HCWs. Of the 269 IPD and OPD patients, 163 (60.6%) were admitted directly to a COVID-19 area, 97 (36.1%) were admitted in a non–COVID-19 area, and the rest (n=9, 3.3%) had stayed in both COVID-19 and non–COVID-19 areas (Table 1).

**Table 1:** Distribution of patients who tested positive for COVID-19 in the hospital from March to August 2020.

Types of patients and areas			Patients, n (%)
**Type of patient (n=360)**			
	Inpatient department patients	240 (66.7)	
	Outpatient department patients	29 (8)	
	Health care workers	91 (25.3)	
**Type of area (excluding staff; n=269)**			
	COVID-19 area	163 (60.6)	
	Non–COVID-19 area	97 (36.1)	
	Both	9 (3.3)	

The CTT identified 3411 HCWs who were exposed to any COVID-19 case in the hospital. After risk categorization, 26.1% (890/3411) of HCWs were identified as high-risk contacts, and 73.9% (2521/3411) were identified as low-risk contacts. Within 14 days of exposure to a COVID-19 case, 34 out of the 890 high-risk contacts (3.8%; 95% CI 2.7%-5.2%) and 48 out of the 2521 low-risk contacts (1.9%; 95% CI 1.4%-2.5%) tested positive for SARS-CoV-2 infection. However, among the low-risk contacts, only symptomatic HCWs were tested, and the test positivity rate among the symptomatic low-risk contacts was 48 out of 1583 (3.03%; 95% CI 2.24%-4.00%).

The mean number of high-risk contacts was 15.8 (SD 18.3) when a COVID-19 case was admitted in a non–COVID-19 area and 4.0 (SD 5.6) when the COVID-19 case was an HCW. The mean number of high-risk contacts per patient was <1 if a patient was admitted in a COVID-19 area or was provided with services on an outpatient basis. The difference in the mean numbers of high-risk contacts among the different groups was statistically significant (*P*<.001; Table 2).

**Table 2:** Comparison of the average number of high-risk and low-risk contacts, with respect to the type of index case, in the hospital from March to August 2020.

Types of patients and areas				Number of cases		Number of contacts, mean (SD)		*t* test^a^ (df) or ANOVA^b^ test (df)			*P* value	
**Type of patient**												
	**High-risk contact**								4.741 (2)^c^			.009
		Inpatient department patients	240		6.61 (13.895)							
		Outpatient department patients	29		0.22 (0.698)							
		Health care workers	91		4.02 (5.653)							
	**Low-risk contact**								8.527 (2)^c^			.002
		Inpatient department patients	240		10.81 (11.754)							
		Outpatient department patients	29		3.07 (2.541)							
		Health care workers	91		8.12 (6.789)							
**Type of area**												
	**High-risk contact**								−10.853 (258)^d^			<.001
		COVID-19 area	163		0.27 (1.207)							
		Non–COVID-19 area	97		15.84 (18.268)							
	**Low-risk contact**								−7.803 (258)^d^			<.001
		COVID-19 area	163		5.93 (5.544)							
		Non–COVID-19 area	97		16.19 (15.188)							

^a^A 2-tailed unpaired *t* test.

^b^ANOVA: analysis of variance.

^c^Analysis of variance test value.

^d^2-tailed unpaired *t* test value.

**Figure 3:**
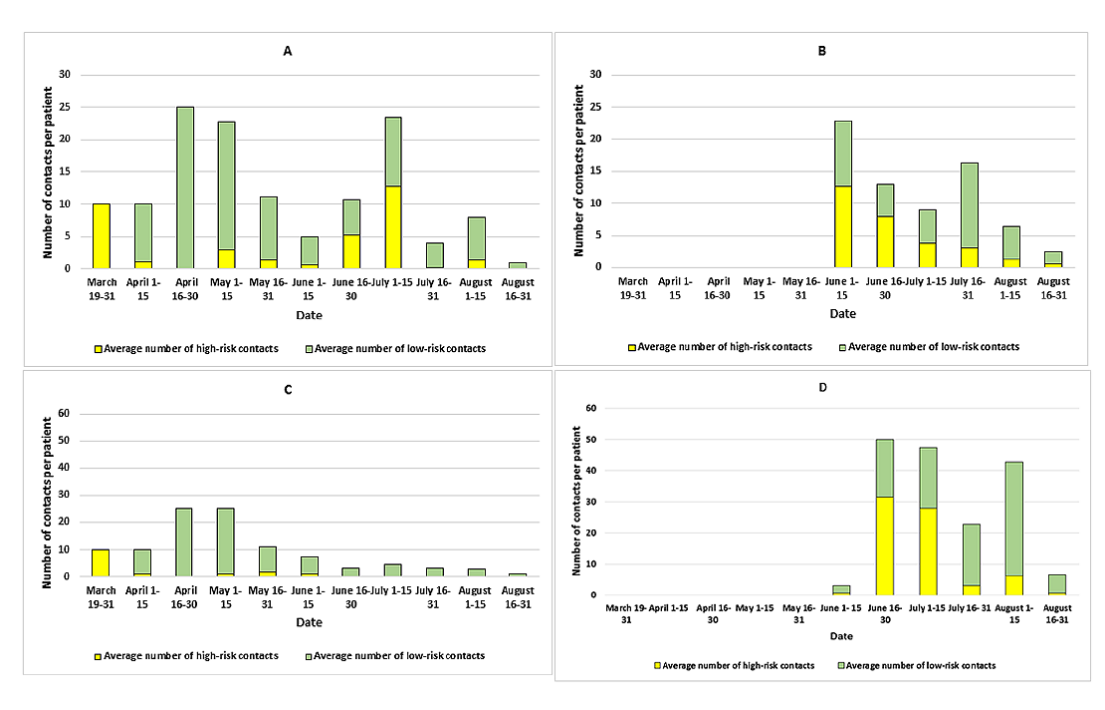
A: Average number of contacts when the COVID-19 case was an admitted patient (March to August 2020). B: Average number of contacts when the COVID-19 case was a health care worker (March to August 2020). C: Average number of contacts when the COVID-19 case was admitted in a COVID-19 area (March to August 2020). D: Average number of contacts when the COVID-19 case was admitted in a non–COVID-19 area (March to August 2020).

A significant decline in the mean number of high-risk contacts was reported over the study period (*P*=.003). In cases where an HCW was the index case, the mean number of high-risk contacts decreased from 12.7 (during June 1 to June 15, 2020) to 3.7 during July 1 to July 15, 2020, and to 0.62 during August 16 to August 31, 2020. The first case of an HCW of the hospital testing positive for COVID-19 occurred on June 2, 2020 (Figure 3). Similarly, in COVID-19 areas, the mean number of high-risk contacts was 10.0 until March 31, 2020, and these contacts involved the only patient who was admitted in a COVID-19 area at that time. Afterward, the mean number of high-risk contacts decreased to 1.0 during April 1 to June 15, 2020, and to <0.1 from June 15, 2020, onward. In the non–COVID-19 area, the mean number of high-risk contacts decreased from 31.5 (during June 16 to June 30, 2020) to 3.0 during July 16 to July 31, 2020, and to 0.7 during August 16 to August 31, 2020 (Figure 3).

Interviews with HCWs, which were conducted during contact tracing, revealed that common causes for a high-risk exposure during the provision of clinical care were the inadequate use of PPE and the nonpracticing of hand hygiene measures after having direct contact with a patient. Further, HCWs who tested positive for COVID-19 indicated that social interactions during meals and at nursing stations during duty hours, handover, travel, and the act of staying together were major contributing factors (24/91, 26%).

## Discussion

### Summary of Results

Our forward contact tracing of 360 COVID-19 cases, who were either patients or HCWs, resulted in the identification of 3411 exposures. After risk stratification, 26.1% (890/3411) of HCWs were categorized as high-risk contacts, and 73.9% (2521/3411) were categorized as low-risk contacts. Of the 890 high-risk contacts and 2521 low-risk contacts, 34 (3.8%) and 48 (1.9%), respectively, tested positive for SARS-COV-2 infection. We also observed a gradual decline in the average number of high-risk contacts over a period of time. HCWs were more likely to be exposed to SARS-CoV-2 infection when it was diagnosed among HCWs and patients who were admitted in a non–COVID-19 area.

### Comparison With Existing Literature

A few studies from India have also reported similar proportions of high-risk contacts after risk stratification. According to a study conducted by Agarwal et al [18], in a hospital located in the eastern part of India, 7/28 (25%) of the COVID-19–exposed HCWs were high-risk contacts. However, this study had a small sample size. Another study, which was conducted by Kaur et al [17] in a hospital located in the northern part of India, reported that only 14.5% of the COVID-19–exposed HCWs were categorized as high-risk contacts. Our study reported a COVID-19 test positivity rate of 3.8% (34/890) among high-risk contacts, while the study conducted by Kaur et al [17] observed a higher test positivity rate (7.1%). In the study by Kaur et al [17], most of the COVID-19 cases (almost 85%) were HCWs, unlike in our study, where 25.3% (91/360) of COVID-19 cases were HCWs. Moreover, the higher COVID-19 test positivity rate in the Kaur et al [17] study could have been due to having more stringent criteria for stratifying HCWs into the high-risk category, as the proportion of high-risk contacts among HCWs was 14% in their study and 26.1% (890/3411) in our study. Another study conducted by Blain et al [19] reported a high COVID-19 test positivity rate among health care personnel (23.5%), but the study was only conducted for 3 COVID-19–positive cases. In our study, 1 index case had 10 high-risk contacts on average, while a study conducted by Vera et al [20] in Switzerland reported 21 high-risk contacts, which was much higher than the number reported in our study. The study from Switzerland was based on just 1 initially undiagnosed COVID-19 case.

We also observed a clear difference in the COVID-19 test positivity rates between high-risk contacts and low-risk contacts (34/890, 3.8% vs 48/2521, 1.9%), which demonstrated the effectiveness of the risk stratification strategy. However, among the low-risk contacts who were tested, the test positivity rate was 3.03% (48/1583). Due to the very high number of contacts, all of the high-risk contacts and only the symptomatic low-risk contacts were tested. Moreover, low-risk contacts could have been exposed to SARS-CoV-2 infection outside of the hospital because, unlike high-risk contacts, they were not quarantined and continued to work. The effectiveness of contact tracing was observed in previous studies [11,14,21].

The mean number of high-risk contacts was highest when a patient was admitted in the non–COVID-19 area (number of contacts: mean 15.8) rather than when a patient was admitted in a COVID-19 area (number of contacts: mean 0.27). The mean number of high-risk contacts was higher in non–COVID-19 areas probably because the recommended level of protection in non–COVID-19 areas is different from that of COVID-19 areas. Similarly, HCWs’ attitudes toward following the protocol might be better in COVID-19 areas due to the higher perceived risk. In COVID-19 areas, HCWs were completely equipped with PPE. In the non-COVID-19 area however, they were only equipped with surgical masks, N95 masks, and gloves, as per the guidelines proposed by the MoHFW, WHO, and CDC, and admitted patients were not suspected of SARS-CoV-2 infection [22-24]. Thus, stringent infection prevention and control measures also need to be adopted in areas where patients who are not suspected of SARS-CoV-2 infection are admitted. Similarly, the number of high-risk contacts was higher when the COVID-19 case was an admitted patient rather than when such cases were HCWs and outpatients. In the IPD, the HCWs perform their duties in a shift-wise manner (3 shifts in 1 day) and perform procedures. In the OPD however, HCWs only have 1 shift per day, and this can result in a fewer number of exposures. Further, the large number of high-risk contacts among COVID-19–exposed HCWs was also the result of social mixing with colleagues during duty time and in residential areas. The importance of workplace social distancing and contact tracing was mentioned in studies conducted by Ahmed et al [25] and Kretzschmar et al [26]. In a community-based study from the United States, the mean number of contacts was 2.4, whereas in our study, it was as high as 10.81 for IPD patients and as low as 0.22 for OPD patients [27]. The reason for this could be the difference in study settings. Our study was conducted in a tertiary care hospital, whereas the Miller et al [27] study was conducted among community participants.

There was also a significant reduction in the number of high-risk contacts for all categories of COVID-19 cases (ie, cases in COVID-19 areas, cases in non–COVID-19 areas, IPD cases, OPD cases, and HCW cases) over the study period (*P*=.003). This reflects the timely modification of infection control measures, the strict implementation of PPE protocols, and the effectiveness of providing infection control–related training to HCWs in the hospital. Similar results were observed in a study that was conducted by Hidayat et al [28] in an Indonesian COVID-19 referral hospital, where the secondary attack rate among HCWs declined from 20.1% to 3.7% over time [28]. However, at the same time, there was a decline in the total number of COVID-19–exposed contacts (low-risk and high-risk contacts combined). This might be indicative of either contact tracing activity–related fatigue in both the CTT and the departments where COVID-19 cases were detected or human resource rationing (this included the modification of duty rosters and fewer rotations of HCWs among different units), which was increasingly performed as the pandemic progressed. Thus, the implementation of strict infection prevention and control measures, PPE protocols, and continuous contact tracing can play a role in mitigating the shortage of human resources. The effectiveness of contact tracing was mentioned in a study by Stuart et al [29].

The CTT also provided regular feedback (based on inquiries from COVID-19–exposed HCWs) to the hospital administration to augment infection control measures, identified areas in which frequent breaches in protocols occurred, and suggested a mechanism for reducing the number of exposures to COVID-19. Apart from quarantine, regular feedback–based action might have helped to reduce the number of exposures to SARS-CoV-2 infection in the hospital.

### Strengths

Since multiple strategies were used, such as visiting the clinical area, conducting personal interviews with the HCWs, reviewing medical records, and viewing CCTV footage, we believe that all of the possible contacts were listed, tracked, and categorized properly, as these strategies were performed by trained personnel and verified by experts. Thus, the quality of the data was expected to be satisfactory. Testing for COVID-19 was performed in an Indian Council of Medical Research–approved testing center via RT-PCR, which is considered to be the gold-standard test. All high-risk cases were continuously monitored for 14 days after their most recent exposure to SARS-CoV-2 infection, and COVID-19 testing was performed on the seventh day.

### Limitations

The categorization of risk was based on the histories of the contacts, which may have increased the chances of social desirability bias affecting our results. Our data might have included misinformation, as hospital staff might have deliberately wanted to be categorized as high-risk contacts, so that they could be quarantined for 14 days and still be fully paid. There was also a chance that HCWs recalled incorrect information. Sometimes, the HCWs failed to remember patients’ SARS-CoV-2 infection status and their own PPE status during patient care. Further, low-risk contacts were not routinely tested unless they were symptomatic. Therefore, we could have missed some cases, as many COVID-19 cases remain asymptomatic or paucisymptomatic.

### Conclusions

Contact tracing and risk stratification were effective and helped to reduce the number of HCWs requiring quarantine. There was a decline in the number of high-risk contacts during the study period. This indicates the role of the implementation of hospital-based COVID-19–related infection control strategies. The findings obtained during contact tracing might also be beneficial for developing appropriate and strategic infection control measures. The contact tracing and risk stratification approaches that were designed in this study can also be implemented in other health care settings.

## Abbreviations

AIIMS: All India Institute of Medical Sciences
CCTV: closed-circuit television
CDC: Centers for Disease Control and Prevention
CTT: contact tracing team
HCW: health care worker
IPD: inpatient department
MoHFW: Ministry of Health and Family Welfare
OPD: outpatient department
PPE: personal protective equipment
RT-PCR: reverse transcription-polymerase chain reaction
WHO: World Health Organization
